# Nucleoside reverse transcriptase inhibitors (NRTIs) induce proinflammatory cytokines in the CNS via Wnt5a signaling

**DOI:** 10.1038/s41598-017-03446-w

**Published:** 2017-06-23

**Authors:** Ting Wu, Juan Zhang, Mingxing Geng, Shao-Jun Tang, Wenping Zhang, Jianhong Shu

**Affiliations:** 1 College of Life Science, Zhejiang Sci-Tech University, Hangzhou, 310018 China; 2 Department of Neuroscience and Cell Biology, University of Texas Medical Branch, Galveston, TX 77555 USA

## Abstract

HAART is very effective in suppressing HIV-1 replication in patients. However, patients staying on long-term HAART still develop various HIV-associated neurological disorders, even when the viral load is low. The underlying pathogenic mechanisms are largely unknown. Emerging evidence implicated that persistent neuroinflammation plays an important role in NeuroAIDS. Although residual virus or viral proteins are commonly thought as the causal factors, we are interested in the alternative possibility that HAART critically contributes to the neuroinflammation in the central nervous system (CNS). To test this hypothesis, we have determined the effect of NRTIs on the expression of proinflammatory cytokines in the various CNS regions. Mice (C57Bl/6) were administered with AZT (Zidovudine 100 mg/kg/day), 3TC (Lamivudine 50 mg/kg/day) or D4T (Stavudine 10 mg/kg/day) for 5 days, and cortices, hippocampi and spinal cords were collected for immunoblotting. Our results showed that NRTI administration up-regulated cytokines, including IL-1β, TNF-α and IL-6 in various CNS regions. In addition, we found that NRTIs also up-regulated Wnt5a protein. Importantly, BOX5 attenuated NRTI-induced cytokine up-regulation. These results together suggest that NRTIs up-regulate proinflammatory cytokines via a Wnt5a signaling-dependent mechanism. Our findings may help understand the potential pathogenic mechanisms of HAART-associated NeuroAIDS and design effective adjuvants.

## Introduction

Human immunodeficiency virus-1 (HIV-1) was identified as the etiologic pathogen for acquired immunodeficiency syndrome (AIDS) over three decades ago^1^. About 35 million people have died of HIV-1 infection, and there are around 36 million people living with HIV^2^. Although there is still no cure for HIV-1 infection, the highly active antiretroviral therapy (HAART, a.k.a. combined antiretroviral therapy, cART) has been proved to be a very effective therapy for inhibiting the viral replication, significantly decrease HIV-associated mortality and morbidities, and become the standard treatment for HIV patients^3^.

Despite its efficiency in suppressing HIV-1 viral load to a very low level, long-term HAART is associated with various detrimental effects. Among the critical HAART side-effects are the damages in the nervous system^4, 5^. Convergent evidence suggests that the prevalence of HIV-associated neurological disorders (HAND) in HIV patients on HAART remains high^6, 7^. HAND in post-HAART era significantly affect the quality of life of HIV patients and may directly contribute to them on-adherence to treatment. However, the potential mechanism by which HAART contributes to HAND is still poorly understood, and interventions are not available.

Neurotoxicity is a suggested mechanism by which HAART could contribute to HAND. Progressive neuron loss was reported in HIV patients on HAART^8^. Antiretroviral drugs also led to neuronal damage and death in animal models^9^. Neurotoxicity appears to associate with major types of antiretroviral drugs in HAART, including nucleoside reverse transcriptase inhibitors (NRTIs), non-nucleoside reverse transcriptase inhibitor (NNRTI) and protease inhibitors (PI)^10–13^.NRTIs are the backbone in current HAART, and ample evidence indicates NRTI-associated neurotoxicity in both peripheral nervous system (PNS) and CNS^14–17^, is probably contributed by their mitochondrial toxicity^18–20^.

Chronic neuroinflammation is implicated in various neurological diseases, including HAND^8, 21–23^. A consistent finding in the postmortem biopsies of HIV patients is neuroinflammation, as indicated by the presence of activated microglia and up-regulated pro-inflammatory cytokines^24^.HIV infection and toxic viral proteins such as gp120 and Tat are commonly thought as the cause of neuroinflammation in HIV patients. Indeed, the activity of gp120 and Tat in inducing neuroinflammation has been demonstrated in cultured glial cells^25–27^ and animal models^28–31^. However, the potential contribution of HAART drugs to the manifestation of persistent neuroinflammation has not been conclusively tested. Because HIV patients usually stay on long-term HAART, this question is clinically relevant.

In this study, we test the hypothesis that long-term administration of NRTIs to mice induces neuroinflammation. We measured the expression level of IL-1β, TNF-α and IL-6 in different CNS regions from mice that were administered with AZT (Zidovudine 100 mg/kg/day), 3TC (Lamivudine 50 mg/kg/day) or D4T (Stavudine 10 mg/kg/day) for 5 days by western blotting. Our results showed that NRTIs up-regulated the cytokines in CNS, and that Wnt5a signaling played a critical role in NRTIs-induced cytokine up-regulation.

## Result

### NRTIs up-regulate the expression of inflammatory cytokines in the CNS

Persistent neuroinflammation is considered to contribute to the development of HAND^32–34^. As HAART is the currently common treatment to suppress HIV replication in patients, we wanted to determine the potential effect of NRTIs, the essential components in HAART, on neuroinflammation in the CNS. Mice (C57Bl/6, males, 6–8 weeks) were subcutaneously injected with AZT (100 mg/kg/day), 3TC (50 mg/kg/day) or D4T (10 mg/kg/day) for 2, 5, 10, or 14 days and CNS tissues including cortices, hippocampi and spinal cords, were collected at the end of NRTIs treatment. Western blotting analysis was performed to determine the expression levels of IL-1β, TNF-α and IL-6. Preliminary experiments indicated an increase of the cytokines was already evident at day 5 after NRTIs administration. Thus, we focused our analysis on this time point to save animals.

**Figure 1:**
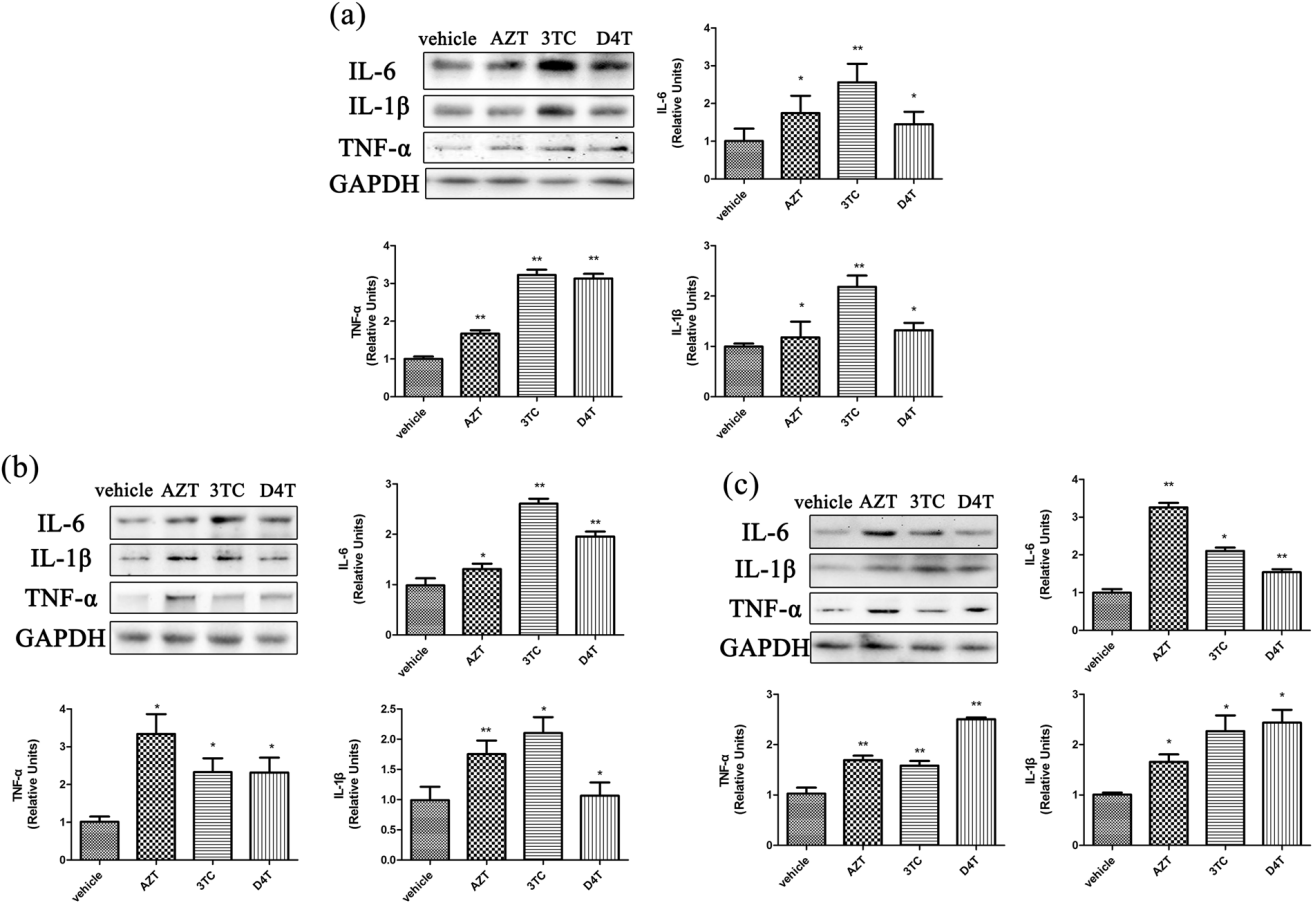
NRTIs up-regulate the expression of inflammatory cytokines in the CNS. Protein levels of cytokine in cortex (**a**), hippocampus (**b**) and spinal cord (**c**) treated with NRTIs for 5 days. Datas presented in graphs are means ± SEM from 5 mice per group *p < 0.05, **p < 0.01, ***p < 0.001vs vehicle.

As shown in Fig. 1, IL-6, TNF-α and IL-1β in cerebral cortices, hippocampi and spinal cords were significantly increased after NRTIs treatment (Fig. 1). Among the NRTIs,3TC (50 mg/kg/day) showed the most evident effect on cerebral cytokines (IL-1β: 2.18 folds, p < 0.01; TNF-α: 3.22 folds, p < 0.01;IL-6: 2.56 folds, p < 0.01) (Fig. 1a). AZT (100 mg/kg/day), 3TC (50 mg/kg/day) and D4T (10 mg/kg/day) also caused different magnitudes of cytokine up-regulation in hippocampi and spinal cords (Fig. 1b,c). These results suggest that NRTIs induce pro-inflammatory cytokines up-regulation in different regions of the CNS.

### NRTIs up-regulate Wnt5a in the CNS

**Figure 2:**
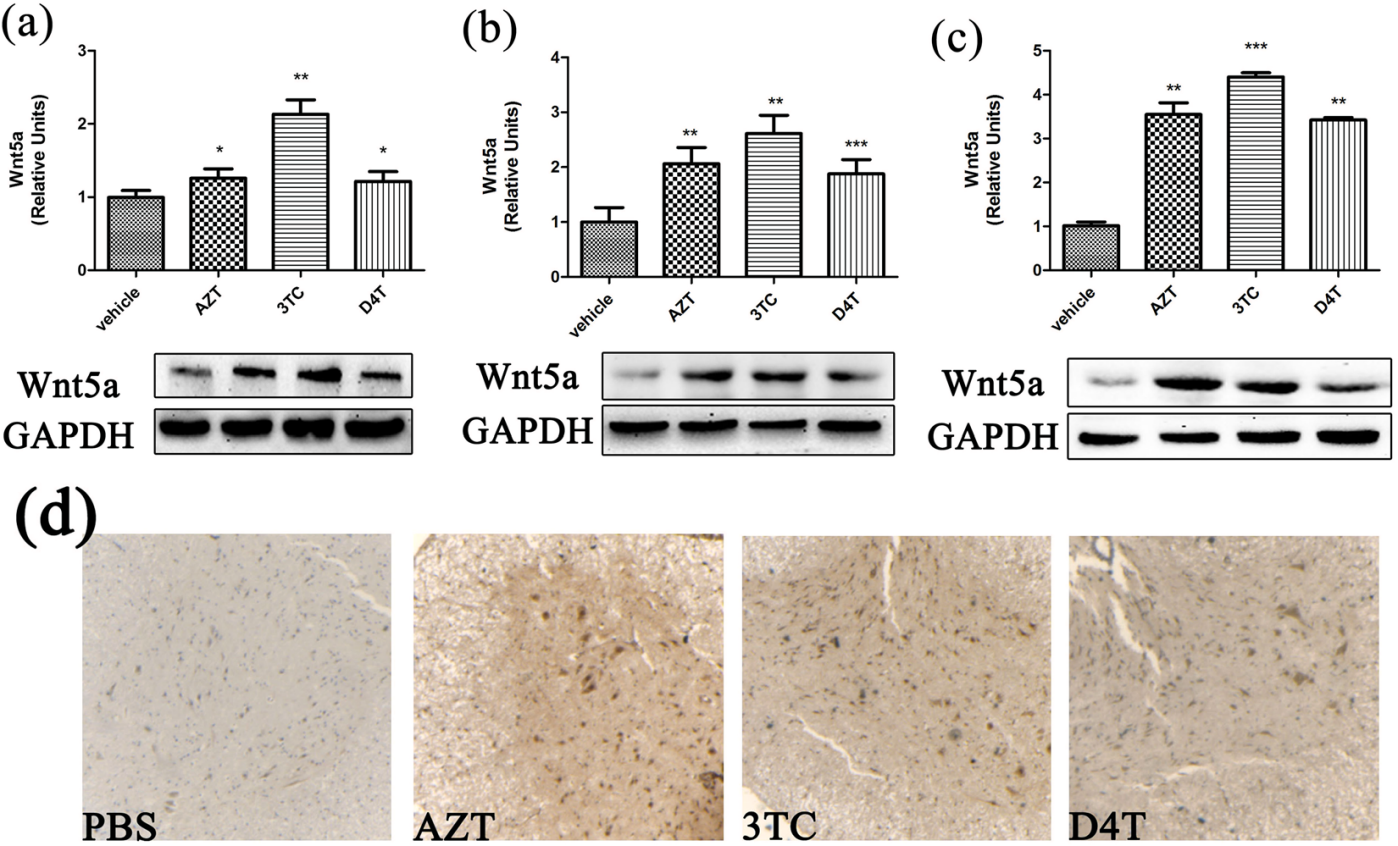
NRTIs up-regulate Wnt5a in the CNS. Protein levels of Wnt5a in cortex (**a**), hippocampus (**b**) and spinal cords (**c**) treated with NRTIs for 5 days. Datas presented in graphs are means ± SEM from 5 mice per group *p < 0.05,**p < 0.01, ***p < 0.001 vs vehicle. (**d**) immunohistochemistry staining of Wnt5a in spinal cords from mice treated with NRTIs.

Wnt5a is emerging as a major inflammatory regulator^35–37^ and is up-regulated in the spinal cord of ‘pain-positive’ HIV patients on HAART^38^. We determined if AZT, 3TC and D4T administration (5 days) up-regulated Wnt5a protein in the cerebral cortex (Fig. 2a), the hippocampus (Fig. 2b) and the spinal cord (Fig. 2c). In the cerebral cortex, Wnt5a was significantly increased in the 3TC group (2.13 fold; p < 0.01). All three NRTIs induced significant Wnt5a increase in the hippocampus (AZT: 2.06 folds, p < 0.01; 3TC: 2.61 folds, p < 0.01; D4T: 1.88 folds, p < 0.001) and the spinal cord (AZT: 3.55 folds, p < 0.01; 3TC: 4.40 folds, p < 0.001;D4T: 3.42 folds, p < 0.01). We also performed immunohistochemistry staining of Wnt5a in the spinal cord and observed that evident increase of Wnt5a after AZT, 3TC or D4T administration (5 days) (Fig. 2d). These datas confirm that Wnt5a is up-regulated in the CNS by NRTIs.

### Wnt5a antagonists attenuate NRTIs-induced cytokine up-regulation in the CNS

**Figure 3:**
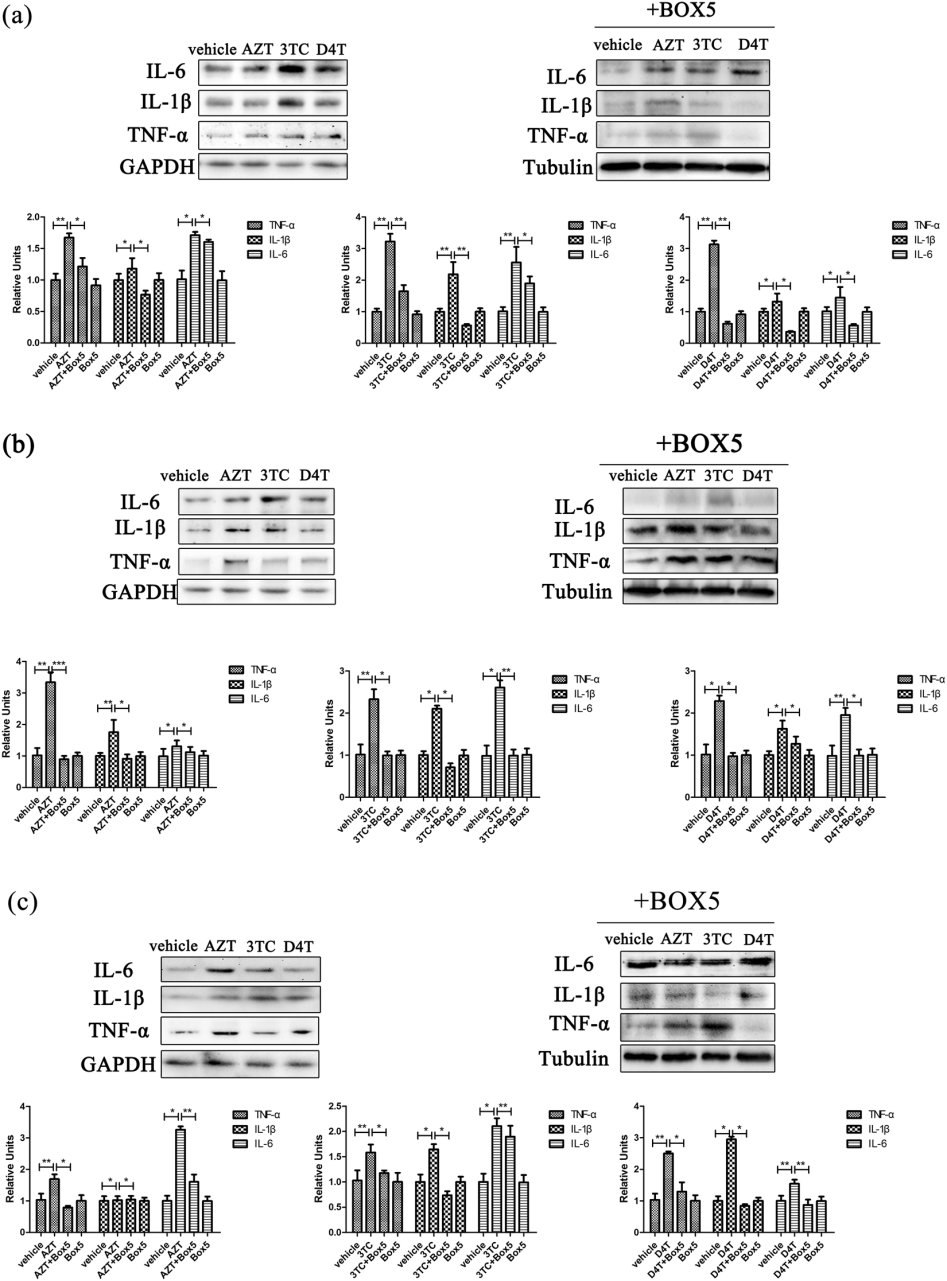
Wnt5a antagonists attenuate NRTIs-induced cytokine up-regulation in the CNS. Protein levels of cytokine in cortex (**a**), hippocampus (**b**) and spinal cord (**c**) treated with NRTIs and BOX5 for 5 days. Datas presented in graphs are means ± SEM from 5 mice per group *p < 0.05, **p < 0.01,***p < 0.001vs vehicle.

**Figure 4:**
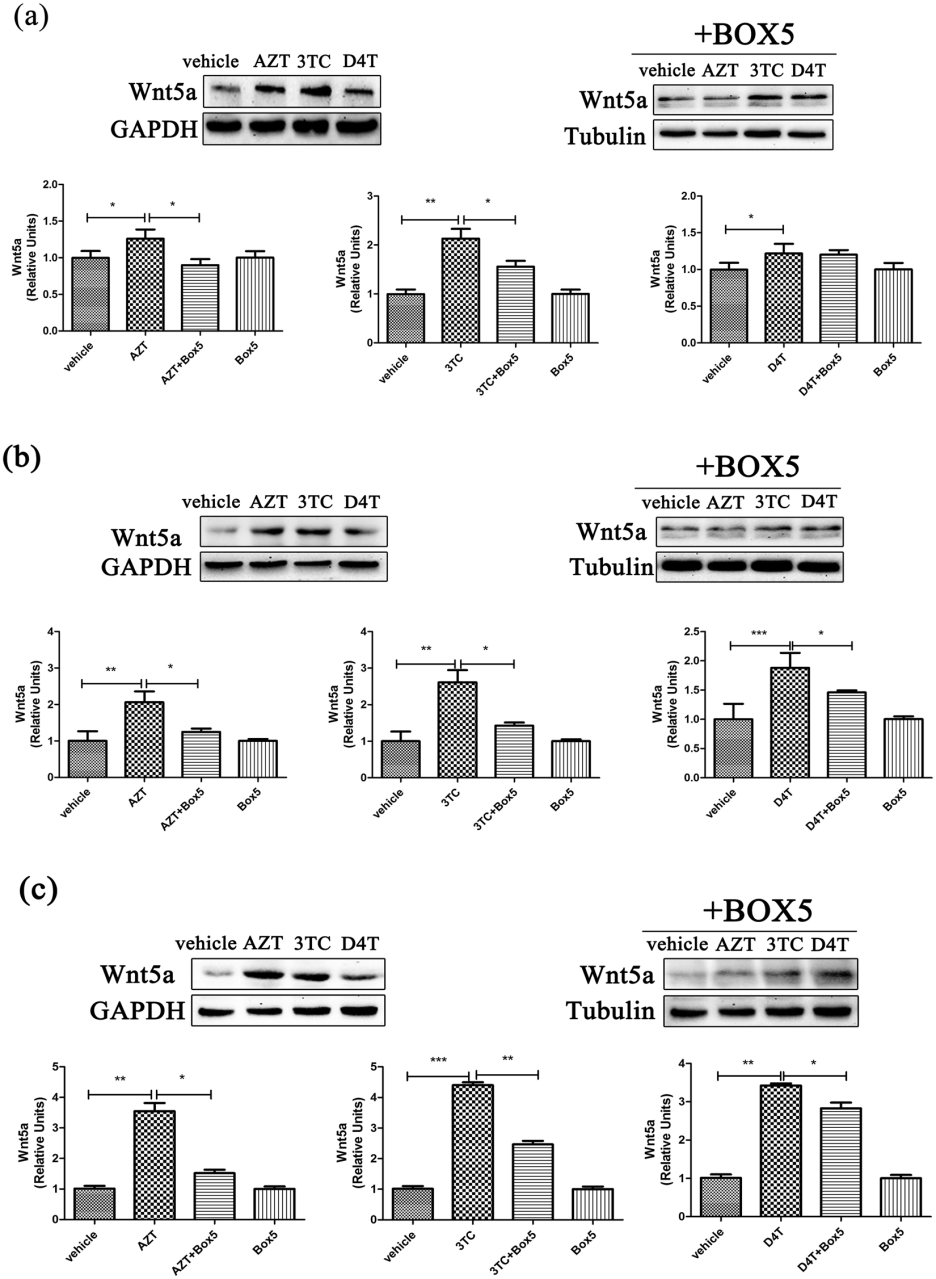
BOX5 inhibited the expression of wnt5a in the CNS. Protein levels of Wnt5a in cortex (**a**), hippocampus (**b**) and spinal cord (**c**) treated with NRTIs and BOX5 for 5 days. Datas presented in graphs are means ± SEM from 5 mice per group *p < 0.05, **p < 0.01, ***p < 0.001vs vehicle.

Wnt5a was reported to regulate HIV-1 gp120-induced cytokine expression in the spinal cord^39^. To investigate the potential role of Wnt5a in NRTI-induced cytokines in the CNS, we determined the effect of Wnt5a antagonist BOX5. BOX5 (10 μg/day)^40^ was administered via the mouse nasal cavity at 3 hours after each NRTI injection. The cerebral cortex, hippocampus and spinal cord were collected for Western blotting after 5 days of drugs administration. We observed that BOX5 administration significantly attenuated NRTIs-induced up-regulation of inflammatory cytokines in the CNS regions (Fig. 3). These results suggest that Wnt5a at least partly mediated the cytokine up-regulation induced by NRTIs. Interestingly, BOX5 treatment also significantly reduced the expression of Wnt5a in the CNS (Fig. 4). This observation indicates that Wnt5a signaling plays a key role in the Wnt5a up-regulation induced by NRTIs.

## Discussion

In this study, we have tested the hypothesis that NRTIs cause neuroinflammation in the CNS. It is well documented that NRTIs can cause neurotoxicity^41, 42^, especially in the peripheral nervous system^11, 43, 44^, but comprehensive investigation on the effect of NRTIs on CNS neuroinflammatory has not been reported. We systematically measure the expression of three cytokines, including IL-1β, TNF-α and IL-6, after the administration of three NRTIs (AZT, 3TC, D4T) in three CNS regions (cortex, hippocampus and spinal cord).Our results show that all three tested NRTIs induce cytokine up-regulation in general, despite that drug-specific effects are observed for different NRTIs. Although it is commonly thought that residual virus and/or viral protein are the causal factors for the commonly observed persistent neuroinflammation in the CNS, our findings indicate that we need to at least include the antiretroviral regimens containing NRTIs as potential critical etiological factor underlying the neuroinflammation in the CNS of HIV patients on HAART. Although some “old’ NRTIs (e.g. AZT and d4T) that are no longer recommended for the first-line treatment are included in the study, this insight may have a general clinically-relevant implication, and is consistent with the reported up-regulation of inflammatory cytokines by other HAART components such as NNRTI and PI in the CNS^45–47^.

Cytokines such as IL-1β, TNF-α and IL-6 are implicated in inflammatory responses associated with various CNS damages^48–51^. Thus, NRTI-induced proinflammatory cytokines revealed in this study may directly contribute to the development of neuroAIDS/HAND^52–56^. In support of this notion, Zheng *et al*. found that TNF-α is involved in neuropathic pain induced by 2′,3′-dideoxycytidine (ddC) in rats^57^. This new understanding may add significant insights into the pathogenesis of neuroAIDS from the perspective of antiretroviral therapy. Flower *et al*. reported an anti-inflammatory activity of NRTIs (e.g. AZT and d4T) in primary human retinal pigment epithelium cells or Raji/TK+ cells^58^. It will be interesting to examine if NRTIs have pro- and anti-inflammatory effects in different biological systems.

Our results further suggest a Wnt5a-mediated mechanism by which NRTIs up-regulate cytokines in the CNS. Specifically, we show that Wnt5a is also up-regulated by NRTIs (Fig. 2a,b,c), and that a specific antagonist of Wnt5a, BOX5, attenuates the NRTIs -induced cytokine up-regulation (Fig. 3).Although Wnt5a has been suggested to regulate inflammation^59–61^, including neuroinflammation^62, 63^, this study reports for the first time an important role of Wnt5a signaling in the regulation of NRTI-evoked CNS neuroinflammation.

**Figure 5:**
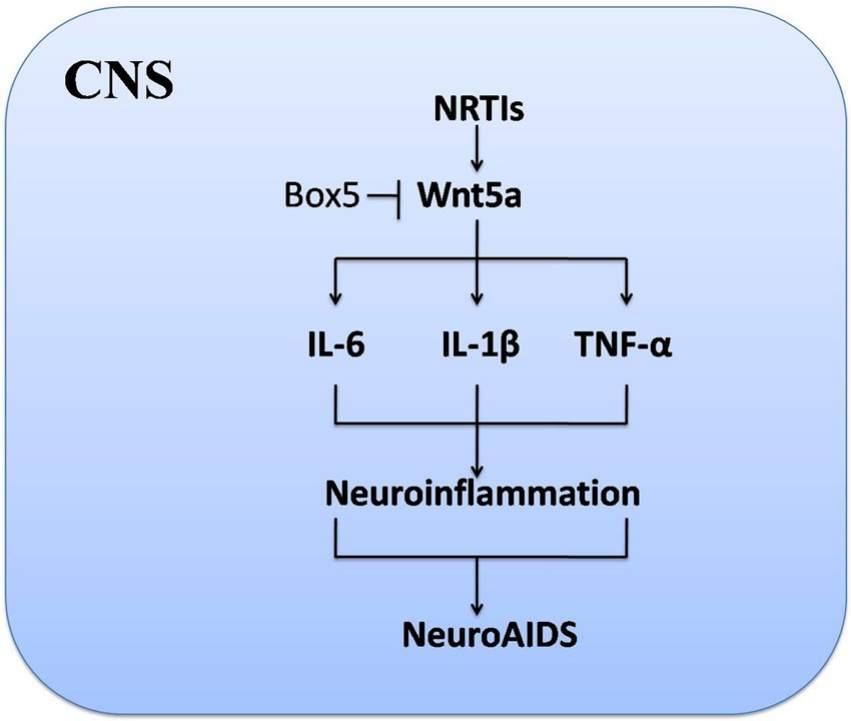
Pathogenetic mechanisms for NRTI-induced neuroAIDS in CNS.

Taken together, our findings suggest that HAART may contribute to neuroAIDS pathogenesis by inducing neuroinflammation in the CNS.Wnt5a signaling probably playscritical role in this pathogenic process by regulating NRTIs-induced cytokines, which could directly contribute to neuroAIDS-related neurodenegeration (Fig. 5).This mechanistic understanding suggests controlling NRTI-induced neuroinflammation as a potential strategy to reduce the risk of neuroAIDS.

## Materials and Methods

### Animals and tissue dissection

All animals (C57BL/6 mice, male, 6–8 weeks) were purchased from Shanghai Ling Chang Biological Technology Co., Ltd. All procedures used in the study were approved by the Zhejiang Sci-tech University Experimental Animal Ethics Committee, and all experimental procedures were carried out in accordance with relevant guidelines and regulations. Specific experimental procedures are described below.

#### Cerebral cortex and hippocampus

Mice were euthanized with inhaled anesthesia ether, and heads were quickly removed and put into phosphate buffer saline (PBS) precooled on ice. The skulls of completely soaked heads were opened and the brains were removed to clean dishes. Cerebral cortices and hippocampi were carefully dissected and frozen immediately inliquid nitrogen. Frozen tissues were stored in a −80 °C refrigerator.Spinal *cord:* The whole spines of euthanized mice were exposed by cutting along the spine skin on the back. After exposure, the spine was cut near the tail cavity. The spinal cord was flashed out with precooled PBS. Collected spinal cords were frozen in liquid nitrogen and stored at −80 °C freezer.

### NRTI Drugs

NRTI drugs: zidovudine (AZT), lamivudine (3TC) and stavudine (D4T) were purchased from Sigma (Sigma-Aldrich). NRTIs were dissolved in PBS to a final concentration of AZT (8 mg/ ml), 3TC (4 mg/ml) and D4T (0.8 mg/ml) and stored at −20 °C.

### Subcutaneous (SC) injection

NRTIs were administered by SC injection. First, the injection site was disinfected with 75% alcohol. Skins of the back of the mouse was gently raised for slowly injecting the drug (0.25 ml/20 g) into the subcutaneous space. After injection, the injection site was pressed gently with alcohol wipes for a moment to prevent drug leakage. Injection volume:0.25 ml/20 g/day.

### Nasal drug administration

Mice were administered with BOX5 (dissolved in PBS to the concentration of 0.5 μg/μL) by nasal dripping as previously described^64^, at 3 hours after NRTI injection. Briefly, a mouse was placed into a fixator tube (a centrifuge tube about the size of the mouse so that the animal can easily claw in). A small hole was made in the bottom of the fixator to expose the nostrils. One person held the animal with the left hand, and gently fixed the mouse head with the right hand to properly expose the nostrils. Another person applied the drug to the nostrils with a 10 μL pipette. Left and right nostrils of mouse were separately administered with BOX5 (10 μL). Drug solution drops were applied to the nostril slowly, usually with one drop every few seconds, and the next drop was applied only after the previous one was completely absorbed. After completing drug application to one nostril, the same procedure was performed to the other nostril. The time interval was about 1 minute. The animal was released from the fixation device after the completion of the whole procedure. Injection volume:10 μL each nostril per day.

### Western blotting and antibodies

Collected tissues were homogenized in cell lysis buffer (Beyotime Institute of Biotechnology), with protease inhibitors (Biotool).The homogenates were centrifugated for 10 min (12,000 g), and protein concentration in the supernatant was measured using the BCA Protein Assay Kit (BIOMIGA). Equal amounts of total protein (40–60 μg) were loaded and separated on 12% SDS-PAGE by electrophoresis (120 V, 90–120 min). Proteins on SDS-PAGE were blotted onto PVDF membranes (100 V, 90–120 min). The membranes were blocked with 5% skim milk dissolved in TBST (20mMTris-HCl,150mMNaCl,0.05%Tween-20) solution for 2 h, and incubated sequentially with primary antibodies in TBST against IL-6 (1:10000, abcamab7737), IL-1β (1:2000, R&D Systems, AF-401-NA), TNF-α (1:1000, abcamab1793), Wnt5a (1:2500, abcamab72583), α-tubulin (1:10000, Proteintech,66031-1-lg) or GAPDH (1:10000, Santa Cruz sc-25778) for 2 h.After washes with TBST3 times for 10 minutes each and the membranes were incubated with goat anti-rabbit IgG (1:30000, abcamab97051) or goat anti-mouse IgG (1:30000 abcamab97023) secondary antibody conjugated with horseradish peroxidase (HRP) in TBST, After washes with TBST3 times for 10 minutes each and the protein bands were visualized by ECL (Advansta). Tubulin and GAPDH were used as loading controls.

### Immunohistochemistry

Immunohistochemistry was performed as described^65^. Briefly, paraffin sections were collected and deparaffinized. Endogenous peroxidase was blocked with 0.3% hydrogen peroxide in methanol. The sections were incubated in EDTA (pH 8.0) buffer at 95–100 °C for 20 min for antigen repair. The pre-treated sections were incubated with primary antibody at room temperature (25 °C) for 0.5–1 h, followed by incubation with secondary antibody for 30 min and staining with DAB (Gene Technology). The slides were lightly counterstained with hematoxylin, rinsed with distilled water, dehydrated in ethanol and mounted.
